# Clinical outcomes after cardiac rehabilitation in elderly patients with and without diabetes mellitus: The EU-CaRE multicenter cohort study

**DOI:** 10.1186/s12933-020-01013-8

**Published:** 2020-03-19

**Authors:** Prisca Eser, Thimo Marcin, Eva Prescott, Leonie F. Prins, Evelien Kolkman, Wendy Bruins, Astrid E. van der Velde, Carlos Peña-Gil, Marie-Christine Iliou, Diego Ardissino, Uwe Zeymer, Esther P. Meindersma, Arnoud. W. J. Van’tHof, Ed P. de Kluiver, Markus Laimer, Matthias Wilhelm

**Affiliations:** 1 Department of Cardiology, Inselspital, Bern University Hospital, University of Bern, Bern, Switzerland; 2 Department of Cardiology, Bispebjerg Frederiksberg University Hospital, Copenhagen, Denmark; 3 Diagram B.V., Zwolle, The Netherlands; 4 Isala Heart Centre, Zwolle, The Netherlands; 5 Department of Cardiology, Hospital Clínico Universitario de Santiago, SERGAS, FIDIS, CIBER CV, University of Santiago de Compostela, Santiago, Spain; 6 Department of Cardiac Rehabilitation, Assistance Publique Hopitaux de Paris, Paris, France; 7 Department of Cardiology, Parma University Hospital, Parma, Italy; 8 Klinikum Ludwigshafen and Institut für Herzinfarktforschung Ludwigshafen, Ludwigshafen, Germany; 9 Department of Cardiology, Radboud University, Nijmegen, The Netherlands; 10 Department of Cardiology, Maastricht University Medical Centre, Maastricht, The Netherlands; 11 Department of Cardiology, Zuyderland Medical Centre, Heerlen, The Netherlands; 12 Department of Diabetes, Endocrinology, Clinical Nutrition & Metabolism (UDEM), Inselspital, Bern University Hospital, University of Bern, Bern, Switzerland

**Keywords:** Cardiac rehabilitation, Cardiovascular risk factors, Exercise capacity, Peak VO_2_, Systolic blood pressure, LDL-C, HbA1c, BMI

## Abstract

**Background:**

The prevalence of patients with concomitant cardiovascular disease and diabetes mellitus (DM) is increasing rapidly. We aimed to compare the effectiveness of current cardiac rehabilitation (CR) programs across seven European countries between elderly cardiac patients with and without DM.

**Methods:**

1633 acute and chronic coronary artery disease (CAD) patients and patients after valve intervention with an age 65 or above who participated in comprehensive CR (3 weeks to 3 months, depending on centre) were included. Peak oxygen uptake (VO_2_ peak), body mass index, resting systolic blood pressure, low-density lipoprotein-cholesterol (LDL-C), and glycated haemoglobin (HbA1c) were assessed before start of CR, at termination of CR (variable time point), and 12 months after start of CR, with no intervention after CR. Baseline values and changes from baseline to 12-month follow-up were compared between patients with and without DM using mixed models, and mortality and hospitalisation rates using logistic regression.

**Results:**

430 (26.3%) patients had DM. Patients with DM had more body fat, lower educational level, more comorbidities, cardiovascular risk factors, and more advanced CAD. Both groups increased their VO_2_ peak over the study period but with a significantly lower improvement from baseline to follow-up in patients with DM. In the DM group, change in HbA1c was associated with weight change but not with change in absolute VO_2_ peak. 12-month cardiac mortality was higher in patients with DM.

**Conclusions:**

While immediate improvements in VO_2_ peak after CR in elderly patients with and without DM were similar, 12-month maintenance of this improvement was inferior in patients with DM, possibly related to disease progression. Glycemic control was less favourable in diabetic patients needing insulin in the short- and long-term. Since glycemic control was only related to weight loss but not to increase in exercise capacity, this highlights the importance of weight loss in obese DM patients during CR.

*Trial registration* NTR5306 at trialregister.nl; trial registered 07/16/2015; https://www.trialregister.nl/trial/5166

## Background

Type 2 diabetes mellitus (T2DM) is a common comorbidity in patients with cardiovascular disease, particularly in elderly patients, since many risk factors are shared between the two diseases, and DM increases the risk for cardiovascular disease (CAD) [1–4].

A recent position paper of the European Association of Preventive Cardiology [5] proposed that exercise training in cardiovascular patients with DM improves glycemic control [6, 7] and may contribute to reducing dyslipidaemia and blood pressure [8]. In elderly patients with T2DM, studies have commonly employed resistance training, in an attempt to address age-related muscle loss and fat infiltration into muscle tissue, which is a common problem in these patients [9]. These studies have not only been successful at improving muscle force but also glycemic control [10, 11]. In a Chinese study in free-dwelling elderly T2DM subjects, exercise was found to be associated with better glycemic control compared to inactive subjects particularly in those with greater waist circumference, high fasting blood glucose and high triglycerides levels [12]. Clinical benefits of exercise-based cardiovascular rehabilitation (CR) programmes have been assessed in younger diabetic populations [13–17] with some studies finding comparable improvements in cardiovascular risk factor management [13, 14, 16, 17], and some finding smaller benefits of CR [15] in patients with DM compared to non-diabetic patients. One retrospective study of the nineties following up patients after CR found mortality, and in particular cardiovascular mortality, to be increased in diabetic compared to non-diabetic patients [18]. However, no studies on clinical benefits of CR or hard outcomes have focussed on an elderly population with DM, and only one very small study has investigated outcomes at 1-year follow-up [19].

The aim of the present study was to compare the improvement in exercise capacity (peak oxygen uptake, VO_2_ peak) with CR programmes offered at the different centres who participated in the EU-CaRE study in elderly cardiovascular patients between patients with and without DM. Further, we were interested to assess whether glycemic control and dyslipidemia were affected by number of attended exercise training sessions (which depended on centre as well as on patient compliance), increase in exercise capacity or weight loss in diabetic patients. Lastly, we compared cardiac and all-cause mortality as well as major adverse cardiac events between patients with and without DM.

## Methods

The EU-CaRE observational study was a study on existing CR programmes provided to elderly cardiac patients at eight European centres, namely Bern, Copenhagen, Ludwigshafen, Paris, Parma, Nijmegen, Santiago de Compostela and Zwolle. The CR program offered at each centre has been described previously [20]. In brief, centres offered between 10 and 36 endurance training sessions on cycling ergometers, mostly as continuous moderate intensity exercise, except for two centres who performed their cycling training as high-intensity interval training. Four of the eight centres also added between 15 and 24 sessions of resistance training. CR programmes lasted between 3 weeks and 3 months. All centres offered dietary counseling. After CR, patients were given guideline based recommendations for physical activity [21].

### Study population

The study population and baseline data have been reported previously [22, 23]. Briefly, patients age 65+ with recent acute coronary syndrome (ACS), chronic coronary artery disease (CAD) with or without revascularization (coronary artery bypass grafting, CABG, and percutaneous coronary intervention, PCI) as well as patients with surgical or percutaneous treatment for valvular heart disease (VHD) participating in CR were included. Patients were assessed before commencing CR (T0), after completing the CR program (T1), and 1 year after completion of CR at 1-year follow-up (T2).

The study was approved by all relevant medical ethics committees, registered at trialregister.nl (NTR5306 and NTR5308) and funded by the European Union’s Horizon 2020 research and innovation program under grant agreement number 634439 and by the Swiss State Secretariat for Education, Research and Innovation for the Swiss consortium partner. The participants gave written informed consent before they were included in the study.

### Data collection

Recorded information included demographics, index event, socioeconomic factors, medical history including co-morbidities and cardiovascular risk factors, lifestyle, and clinical information such as weight, body mass index (BMI), blood pressure (BP), resting heart rate and exercise capacity, medication, and patient reported outcomes such as physical activity in terms of number of days per week with at least moderate physical activity of minimally 30 min. Low physical activity was defined as less than 5 days per week with a minimum of 30 min of at least moderate activity. Blood samples were taken non-fasting. The functional assessment consisted of a cardiopulmonary exercise test (CPET) or 6 min walking test (6MWT). Attended number of sessions divided by offered session was considered as compliance. Offered sessions ranged from 10 to 36. Details on the collected data have been provided elsewhere [20, 22].

Patients were grouped according to presence or absence of DM as follows: previous diagnosis with DM, intake of insulin or oral antidiabetics at start of CR, HbA1c at baseline of ≥ 48 mmol/mol.

CPETs were performed on a cycle with an individualised ramp protocol aiming to achieve voluntary exhaustion within 8 to 12 min. Raw data was analysed in the CPET core lab in Bern using MATLAB software from MathWorks^®^. Gas measures were excluded from the analysis in case of suspected mask leakage or equipment failure or if the ramp duration was less than 3 min. In these cases, as well as for 6MWT, peak VO_2_ was calculated with a formula using the maximum Watt [24].

Some of the secondary outcomes were dichotomized with regard to reaching target levels according to current guidelines [25] as follows: systolic BP < 140 mmHg, LDL-C < 1.8 mmol/l or lowering of LDL-C by ≥ 50%, BMI < 30 kg/m^2^ (non-obesity) or lowering body weight by ≥ 5%, and HbA1c in diabetic patients < 53 mmol/mol. Adverse events were recorded by monthly telephone calls and assessed individually by an independent Clinical Event committee. Major Adverse Cardiac Event (MACE) were defined as composite endpoint of all-cause and cardiovascular mortality, acute coronary syndrome, aborted sudden cardiac death and cardiovascular intervention/surgery, hospital admission or emergency visits between T0 and T2.

### Statistical analysis

All statistics were performed with R (Version 3.5.1, R Core Team, 2017).

Baseline parameters, compliance with CR and hard outcome parameters were compared between the patient groups with and without DM by Chi squared test, independent t-tests or Wilcoxon two sample tests as appropriate. Mixed linear models were performed for peak VO_2_ [ml/min/kg] measured at all time points (lme function from package nlme) with patients nested within centres as random factors and the following fixed factors: DM, time point, age, sex, index intervention as well as baseline parameters found to differ between groups, such as BMI, hypertension, hypercholesterolemia, previous ACS, nephropathy, and peripheral arterial disease. Due to multiple testing, alpha level was set at 0.01 for all analyses. The interaction between time and DM was also entered into the model to assess changes over time that differed between groups. The same models were also performed for the following secondary outcome parameters: First ventilatory threshold, ratio ventilation:carbon dioxide output  (VE/VCO_2_) slope, resting heart rate, peak heart rate, heart rate recovery, systolic and diastolic BP, pulse pressure, LDL-C, HDL-C, triglycerides, BMI, HbA1c, and creatinine.

Within the DM group, a robust linear regression model (lmrob function from package robustbase) was performed for change in HbA1c between T0 and T2 and independent variables BMI at baseline, change in weight between T0 and T2, change in absolute peak VO_2_ between T0 and T2, and number of attended exercise training sessions.

Logistic regression models were performed for all-cause and cardiac mortality using the glmer function (from package lme4) with independent factors DM, age, sex, BMI, index intervention (PCI, CABG, valve replacement, stable angina), and previous acute coronary syndrome as fixed factors and centre as random factor.

## Results

### Study population

Of the included 1633 patients, 405 were already diagnosed with DM at T0, of whom 10 were diagnosed with type 1 DM, and in 3 patients data on presence or absence of DM was missing. Of the patients not diagnosed as diabetic at T0, 25 patients had HbA1c levels of ≥ 48 mmol/mol. Consequently, a total of 430 patients were included in the group of patients with DM (26.4%) at baseline and 1200 patients in the group without DM at baseline. Main outcomes of the EU-CaRE study have been published recently [26].

Of the 430 diabetic patients who completed baseline testing at T0, 397 (92.3%) completed an exercise test at T1 and 368 (85.6%) at T2, while in the non-diabetic patients, of the 1200 patients completing T0, 1123 (93.6%) completed T1 and 1086 (90.5%) T2.

With regard to the different centres, the percentage of included patients with DM ranged from 18.8% (Nijmegen), 21.9% (Copenhagen), 23.3% (Paris), 23.9% (Bern), 25.0% (Zwolle and Ludwigshafen), 29.6% (Parma) to 35.8% (Santiago).

Diabetic patients were of comparable age but tended to include fewer females (Table 1). At baseline, BMI was 2 kg/m^2^ higher in patients with DM, which was reflected in a 6 cm greater waist circumference, 4 mm greater skinfold thickness and 1% greater body fat. Patients with DM had lower educational level, were less physically active, had an 8% higher rate of previous ACS, 13% and 12% higher rate of hypertension and hypercholesterolemia, respectively, as well as more than double the prevalence of nephropathy and peripheral arterial disease.

**Table 1:** Baseline characteristics of diabetic and non-diabetic patients

Parameter	Diabetic (430)	Non-diabetic (1200)	p-value
Physical characteristics			
Female sex (%)	82 (19.1)	292 (24.3)	0.031
Age (years)	72.6 ± 5.5	73.0 ± 5.4	0.274
BMI (kg/m^2^)	28.6 ± 4.1	26.6 ± 4.0	0.0000
Waist circumference (cm)	104.2 ± 11.4	98.5 ± 11.2	0.0000
Skinfold thickness (mm)	64.3 ± 23.2	58.7 ± 20.2	0.0000
Body fat (%)	30.5 ± 6.4	29.5 ± 6.2	0.007
Educational attainment			0.0001
Primary education	145 (34.0%)	314 (26.3%)	
Secondary education	209 (49.1%)	579 (47.4%)	
Tertiary education	72 (16.9%)	313 (26.3%)	
Index intervention			0.003
VHD	37 (8.6%)	129 (10.8%)	
CABG	136 (31.6%)	344 (28.7%)	
PCI	218 (50.7%)	670 (55.8%)	
Stable angina	39 (9.1%)	57 (4.8%)	
Cardiovascular risk factors			
Smoking (active/former/never)	44/76/309	110/193/897	0.543
Days with > 30 min physical activity	3.3 ± 2.8	4.0 ± 2.7	0.0000
Previous ACS	104 (24.3%)	200 (16.7%)	0.0007
Comorbidities			
Hypertension	333 (77.4%)	775 (64.6%)	0.0000
Hypercholesteremia	326 (76.0%)	770 (64.2%)	0.0000
Family history of CVD	111 (26.0%)	383 (32.0%)	0.024
Nephropathy	57 (13.3%)	67 (5.6%)	0.0000
Chronic heart failure	16 (3.7%)	26 (2.2%)	0.115
Peripheral arterial disease	57 (13.3%)	68 (5.7%)	0.0000
Obstructive sleep apnea	19 (4.4%)	24 (2.0%)	0.012
Anaemia	200 (57.0%)*	484 (49.4%)*	0.018
Atrial fibrillation	34 (7.9%)	79 (6.6%)	0.408
LV ejection fraction < 35%	19 (4.4%)	46 (3.9%)	0.290
Medication			
Insulin	114 (26.5%)		
Oral antidiabetics	253 (58.8%)		
Beta blocker	359 (83.5%)	965 (80.4%)	0.184
Statins	394 (91.6%)	1063 (88.6%)	0.095
ACE inhibitors	198 (46.0%)	609 (50.8%)	0.103
ARBs	110 (25.6%)	198 (16.5%)	0.0000

BMI, body mass index; VHD, valvular heart disease; CABG, coronary artery bypass grafting; PCI, percutaneous intervention; ACS, acute coronary syndrome; CVD, cardiovascular disease; LV, left ventricular; ACE, angiotensin-converting-enzyme; ARBs, angiotensin II receptor blockers

* Values available of only 351 patients with DM and 979 patients without DM

At baseline, 50 diabetic patients (11.6%) were on insulin and oral antidiabetics, 64 (14.9%) received only insulin, and 203 (47.2%) only oral antidiabetics, while 113 (26.3%) had no DM medication. Information on diabetic medication was missing in 10 patients (2.3%). At T1, information on diabetic medication was missing in 22 patients (5.1%). Of patients with available data on DM medication at T1, 88 still had no DM medication (11.6%). At least 18 additional patients were on oral antidiabetics. At T2, information on DM medication was missing in 54 patients with DM (of whom 10 patients had died). Eighty-two patients with DM still had no antidiabetic medication. Diabetic patients were more commonly on Angiotensin II receptor blockers (Table 1).

Median compliance with CR physical training sessions was high overall but lower in patients with DM (94%, IQR 83–100%) compared to those without DM (100%, IQR 87–100%, *p* value from Wilcoxon two sample test 0.006).

### Primary outcome

**Fig. 1:**
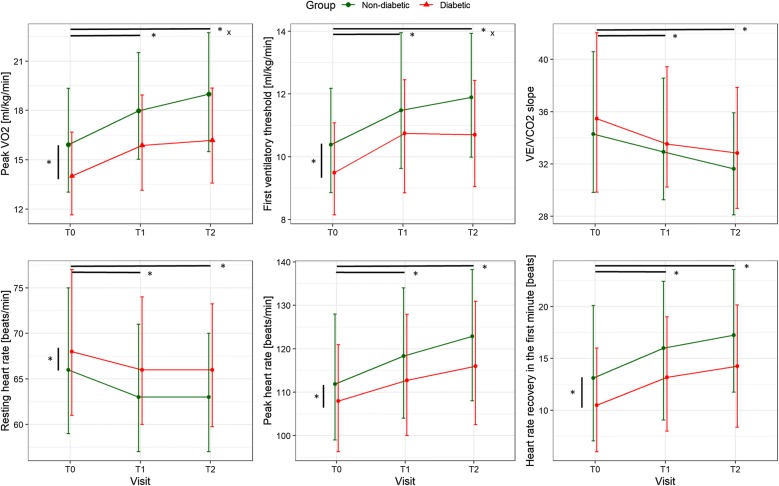
Primary outcome parameter VO_2_ peak and other parameters from cardiopulmonary exercise testing at baseline (T0), end of CR (T1) and 1-year follow-up (T2) in patients with and without DM. Shown are medians and interquartile ranges. Black lines indicate significant differences in the adjusted mixed models with asterisk indicating p ≤ 0.01 for main group or time effects and x for p ≤ 0.01 for group x time interaction effect

In the mixed model adjusted for index intervention, sex, age, BMI, comorbidities and cardiovascular risk factors (as well as mean VO_2_ peak of each patient due to entering patients as random factors), presence of DM significantly and independently reduced VO_2_ peak by 1.46 ml/kg/min. In both groups, VO_2_ peak improved over the course of CR but with a significantly smaller change (− 0.6 ml/kg/min) from T0 to T2 in patients with DM (Fig. 1, top left panel, and Additional file 1: Table S1).

### Secondary outcomes

**Fig. 2:**
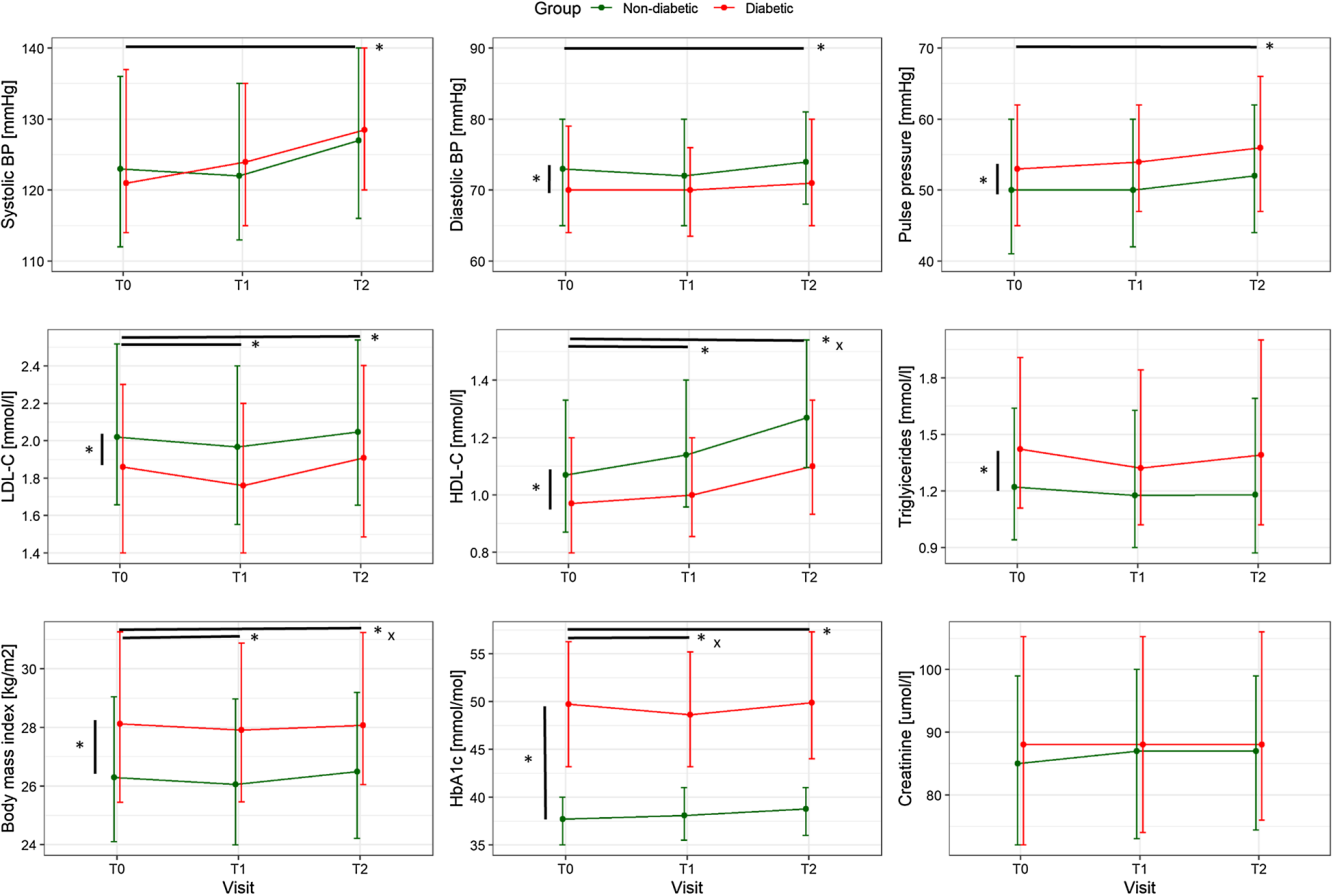
Secondary outcome parameters at baseline (T0), end of CR (T1) and 1-year follow-up (T2) in patients with and without DM. Shown are medians and interquartile ranges. Black lines indicate significant differences in the adjusted mixed models with asterisk indicating p ≤ 0.01 for main group or time effects and x for p ≤ 0.01 for group × time interaction effect

Reported results are from mixed models adjusted for age, sex, BMI, index intervention (surgery vs. non-surgery), comorbidities and cardiovascular risk factors, however, results shown in Figs. 1 and 2 are unadjusted. Similar to VO_2_ peak, the first ventilatory threshold was − 0.48 ml/kg/min lower in diabetic compared to non-diabetic patients, and long-term maintenance was significantly worse in diabetic patients (Fig. 1, top middle panel), while the improvement of VE/VCO_2_ slope over time was not affected by DM (Fig. 1, top right panel). Resting heart rate was overall 2.2 bpm higher in patients with DM but was improved similarly in patients with and without DM by − 3.5 bpm from T0 to T2 (Fig. 1, bottom left panel). Peak heart rate was 3.9 bpm lower in patients with DM, but improved similarly over time by 8.9 bpm at T2 (Fig. 1, bottom middle panel). Consequently, heart rate reserve was reduced by 6.2 bpm in diabetic patients but improved similarly over time by 12.4 bpm at T2. In parallel with heart rate reserve, heart rate recovery was also reduced in diabetic patients by 2.3 bpm and improved similarly to non-diabetic patients by 3.7 bpm at T2 (Fig. 1, bottom right panel).

Patients with DM had comparable systolic BP, significantly lower diastolic BP (by 2 mmHg), and significantly higher pulse pressure (by 3 mmHg), with all BP parameters increasing from T0 to T2 by 3.9 mmHg, 1.4 mmHg and 2.7 mmHg, respectively (Fig. 2, top panels). LDL-C and HDL-C were significantly lower in diabetic patients at all time points compared to non-diabetic patients (Fig. 2 middle left and central panels). LDL-C decreased significantly by a negligible of 0.09 mmol/l between T0 and T1 with no difference between patients with and without DM. At T2, LDL-C had significantly increased from T0 by 0.06 mmol/l. There was a consistent increase in HDL-C over time, however, this increase was significantly smaller at T2 in the DM group. Triglycerides remained stable over time but were higher at all time points in diabetic patients (Fig. 2, middle right panel). BMI decreased significantly but negligibly in both groups by 0.15 kg/m^2^ between T0 and T1, but was significantly higher by 0.15 kg/m^2^ at T2 compared to T0 (Fig. 2, bottom left panel). In the adjusted models, BMI of diabetic patients was 1.7 kg/m^2^ higher than BMI of non-diabetic patients. Unadjusted body weight was approximately 5 kg or 7% of predicted weight higher in DM patients compared to non-DM patients at T0 and T2.

No parameters were found to significantly account for changes in LDL-C within the DM group. However, within the DM group, change in triglycerides was inversely related to weight change, while change in HDL-C was related to change in VO_2_ peak.

**Fig. 3:**
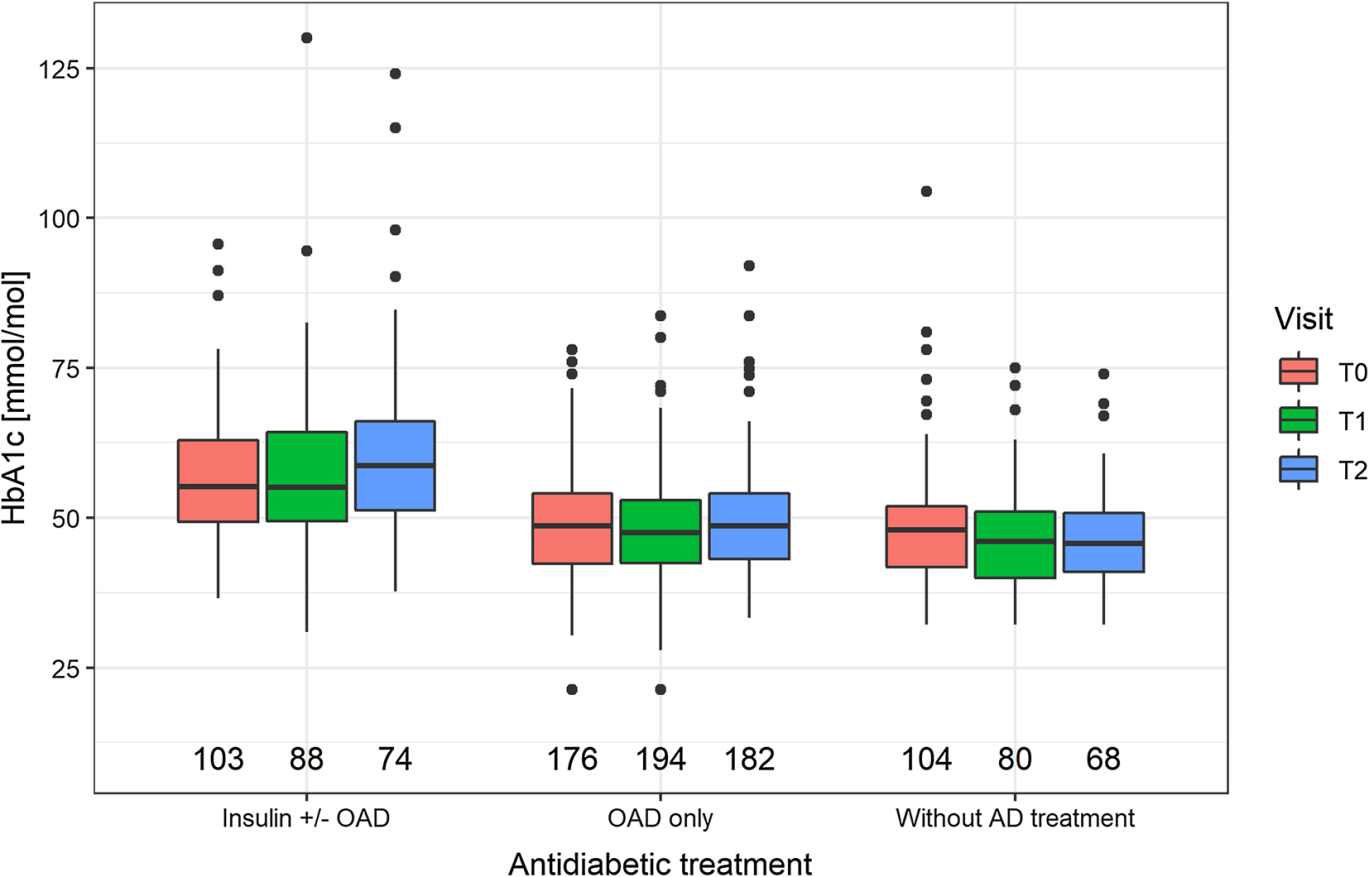
Boxplots of HbA1c levels in diabetic patients at baseline (T0), end of CR (T1) and 1-year follow-up (T2) according to patients with insulin therapy with or without oral antidiabetic medication (OAD, n = 114), OAD without insulin (n = 203) or no antidiabetic treatment (n = 113)

In the adjusted model, HbA1c was on average 12.9 mmol/mol higher in patients with DM compared to those without. From T0 to T1 HbA1c decreased by a median of − 1.0 mmol/mol in patients with DM, but increased by a median of 0.1 mmol/mol in patients without DM (Fig. 2, bottom central panel). From T1 to T2, HbA1c increased by a median of 1.1 mmol/mol in patients with DM, and remained stable in patients without DM. 36.3% of patients with DM had HbA1c ≥ 53 mmol/mol at T0 (data of 47 patients with DM missing), 33.1% at T1 (68 missing) and 46.6% at T2 (104 missing). Amongst patients without DM, 2.2% had HbA1c ≥ 48 mmol/mol at T1 and 2.1% at T2. HbA1c was related to BMI and was 6.0 mmol/mol higher in diabetic patients taking insulin  (Fig. 3). In the model for change in HbA1c between T0 and T2, HbA1c at T0, BMI at T0, insulin, and weight change between T0 and T2 were significant factors, with every 1 mmol/mol higher HbA1c at T0 reducing the increase in HbA1c between T0 and T1 by 0.5 mmol/mol, and for every kg increase in body weight between T0 and T2, HbA1c increased by 0.4 mmol/mol more. Neither number of attended exercise training sessions nor change in VO_2_ peak were significantly related to HbA1c. Patients on insulin increased their HbA1c by 3.4 mmol/mol more than patients without insulin. Of the diabetic patients, 183 (44.4%) attended group dietary counselling sessions and 125 (30.3%) attended individual dietary counselling. However, attending dietary counselling had no effect on changes in HbA1c.

Creatinine was comparable between patients with and without DM and did not change over time (Fig. 2, bottom left panel).

### MACE

All assessed hard outcome parameters tended to be higher in diabetic patients (Table 2). Only cardiac mortality reached statistical significance, however, incidence was low in both groups (2.3% in diabetic and 0.6% in non-diabetic patients). DM was the only significant factor for cardiac mortality also in the adjusted logistic regression models with an odds ratio of 4.4 (99% CI 1.08–18.2).

**Table 2:** Hard outcome parameters at 12 months follow-up for CAD patients. Shown are number of patients (percent of all patients in this group) with event

Parameters	Diabetic patients N = 393	Non-diabetic patients N = 1071	p-value
MACE	71 (18.1)	142 (13.3)	0.026
Cardiac mortality	9 (2.3)	6 (0.6)	0.009
Acute coronary syndrome	9 (2.3)	19 (1.8)	0.672
Hospitalisation for cardiac reason	43 (10.9)	85 (7.9)	0.089
Cardiac related emergency visits	39 (9.9)	77 (7.2)	0.108
Cardiac intervention	39 (9.9)	87 (8.1)	0.325
All cause mortality	9 (2.3)	13 (1.2)	0.209

MACE, major adverse cardiac event

p-values are from Chi squared test

## Discussion

This study compared the short and long-term (12 months) benefits with CR in elderly patients with and without DM. Patients with DM had higher BMI, higher pulse pressure, lower heart rate reserve, a higher prevalence of previous ACS, more comorbidities and a lower VO_2_ peak. VO_2_ peak improved in both groups over the course of the study but 1-year improvement was poorer in patients with DM. Short term changes were significantly better in patients with than in patients without DM for HbA1c, however, there was a comparable long-term increase in HbA1c in both groups.

This is the first study on clinical benefits with CR in exclusively elderly diabetic patients. Our results confirm findings of previous studies in younger populations that have shown comparable clinical benefits of CR between cardiovascular patients with and without DM [13, 14, 16, 17], although some found smaller benefits [15, 27, 28]. Our study showed that benefits with CR may be comparable between elderly patients with and without DM over the duration of CR, but were poorer over 1 year follow-up in diabetic patients with regard to exercise capacity and HDL-C. Only one previous small study has assessed 1-year maintenance of the improvement in exercise capacity by CR [19]. They found it maintained in patients with and without DM, however, that study was based on 12 diabetic (and 25 non-diabetic) patients only. Since the present study did not include a control group (not undergoing CR), we cannot infer whether there was a lesser long-term benefit of CR in patients with compared to those without DM, as the lesser improvement in VO_2_ peak at 1-year in the diabetic patients may have also been due to the natural progression of disease.

### VO_2_

Similar VO_2_ peak values compared to ours were found in a recent single centre study, with similar pre- and post-CR values and similar deficits of diabetic compared to non-diabetic patients [14]. The deficit in VO_2_ peak found in our DM population was also reflected in lower VO_2_ at the first ventilatory threshold and the VE/VCO_2_ slope, consistent with what was documented previously [29, 30]. Likewise, in our diabetic patients compared to those without DM, median peak heart rate was 4 bpm and 7 bpm lower at baseline and 12-month follow-up, respectively, and resting heart rate 2 bpm and 3 bpm higher, resulting in a 6 bpm and 10 bpm lower heart rate reserve, which may have, at least in part, accounted for the lower VO_2_ peak. Chronotropic incompetence has been found to be prevalent in diabetic populations [31, 32], and both, DM and chronotropic incompetence are additive predictors for all-cause mortality [32]. The chronotropic incompetence may, at least in part, also explain the reduced heart rate recovery in our as well as in previous populations with DM [33]. Other factors that potentially contribute to lower VO_2_ peak in patients with DM may be reduced diastolic function [34] and lower oxygen extraction by skeletal muscles [35], with the latter being supported by the higher VE/VCO_2_ slope [36].

A larger improvement of just over 1 MET, corresponding to 28% was found in a similarly large population of diabetic cardiovascular patients after a 6-week CR [14]. A retrospective study in patients who completed a minimum of 7 weeks of a 12-week CR found an improvement of over 1.5 METs in diabetic and non-diabetic patients [17]. In accordance to our results, these studies found similar improvements in diabetic compared to non-diabetic patients, albeit at a lower level of VO_2_ peak. However, all of these studies were based on younger cardiac patients. Our 12-month follow-up assessment revealed that the further improvement after end of CR was reduced in diabetic patients when compared to non-diabetic patients. The poorer long-term development of VO_2_ peak in our elderly diabetic patients was also found previously [37] and may reflect the natural disease progression that is associated with reduced cardiovascular fitness [38]. The previously reported relationship between glycemic control with gain in VO_2_ peak [39] was not found in the present study, however, the assessment of HbA1c may not have allowed us to assess short term changes in glycemic control. Furthermore, the causality and direction of this relationship is unclear.

### Blood pressure

Systolic blood pressure was similar in patients with and without DM, was overall well controlled, but increased significantly at 1-year follow-up in both groups. Due to a lower diastolic BP, diabetic patients had a significantly higher pulse pressure than non-diabetic patients at all time points. This is in contrast to a large German registry study that found a 3 mmHg higher systolic and diastolic BP in diabetic patients compared to non-diabetic CAD patients [13]. The reason for the conflicting results may be the older mean age of the patients of our compared to the German registry study. The association of pulse pressure and arterial stiffness [40] may imply that our diabetic patients had higher arterial stiffness than the non-diabetic patients [40], which has also been suggested in previous studies [41–43]. The reason for this may be early ageing of the vessel properties due to the latent chronic inflammation in patients with DM [44, 45]. A decrease in diastolic pressure as a result of increased arterial stiffness may only become apparent at a more advanced age, as apparent in our population, but not in previously investigated younger populations. Increased pulse pressure has also been found to be associated with increased risk for CAD in patients with DM [46–48]. The relationship between pulse pressure and risk of coronary artery events is J-shaped [46] with an increased risk with higher pulse pressure in the initial disease state. With advancing coronary artery disease, pulse pressure first increases. However, at a more advanced stage with concomitant systolic dysfunction, pulse pressure may drop while the risk for coronary artery events increases even further. Similarly, in a large study on patients with type 2 DM and renal impairment, the association of cardiovascular events, cardiovascular deaths and all-cause mortality with systolic BP was also J-shaped with optimal outcome at a systolic BP of 135–139 mmHg [49]. While endothelial function has been found to improve with exercise in diabetic populations [50], we did not measured this in the present study, however, pulse pressure did not improve neither in the short- nor long-term.

### HbA1c

In line with previous reports, change in HbA1c was inversely related to baseline HbA1c, suggesting either that patients with high HbA1c at T0 were treated more aggressively [6], or that blood loss during surgery may have led to lower levels of HbA1c independently of glycemic control with subsequent recovery [51, 52]. In contrast to previous studies, we did not find a relationship between training volume (number of attended training sessions) or change in VO_2_ peak and change in HbA1c [6]. A reason for this may have been the overall small increases in VO_2_ peak which themselves were not associated to number of training sessions (will be reported elsewhere). In the literature on exercise training in elderly diabetic patients, studies showing a decrease in HbA1c are approximately balanced with studies showing no effect on HbA1c, and those that showed an effect found endurance and resistance training equally effective at lowering HbA1c, with combined training showing the best results [53, 54]. Our study had an observational design and included patients who participated in CR rather than patients randomised to exercise. A randomised exercise study would clearly attract fitter and more motivated elderly patients and not reflect the general elderly CR population. Effects of exercise on HbA1c have been rated as small and comparable to the effects of dietary, drug and insulin treatment [55]. However, treatment effects of drugs can be considerable when baseline HbA1c is high [56]. In our elderly DM population, weight loss was related to better glycemic control. This is in line with a recent meta-analysis, which found that weight loss of > 5% was associated with improved glycemic control [57]. This suggests that weight loss is an important aspect of CR programmes. Nevertheless, exercise training should not be neglected as it reduces the loss of muscle mass [58].

### Triglycerides

In our diabetic group, triglycerides tended to decrease by only -0.05 mmol/l between T0 and T1, which is considerably less than the 0.3 mmol/l reported in a meta-analysis on the effects of 34 exercise training studies in patients with T2DM [8]. Again, our population was most likely older than the general population of patients with DM participating in exercise training studies. Further, reduction of anaemia over time, particularly in surgery patients, may have also increased lipid levels [59], which was confirmed by a significant positive association between the change in haemoglobin and change in triglycerides, masking an effect that therapeutic management may have had.

### LDL-C

LDL-C and HDL-C were found to be lower in diabetic compared to the non-diabetic patients at all time-points. This is in accordance to previous studies, who also found a similar decrease in LDL-C from before to after CR in diabetic as well as non-diabetic patients and consistently lower values of LDL-C in diabetic patients [13, 15]. Lipid lowering drug therapy may have been more aggressive, with a trend for higher prescription rate of statins in patients with DM (92% vs. 89%, p = 0.095) and possibly higher doses of statins. Combination therapy with Ezetimibe was similar in patients with and without DM (7% vs. 6%, respectively). Other studies have also found the reduction in LDL-C to be the same in diabetic compared to non-diabetic patients [17, 27].

### BMI

In line with previous studies, only negligible changes in body weight were found in this study [8, 17]. However, some studies found larger changes in BMI compared to our study with smaller or comparable changes between patients with and without DM [15, 27]. Despite significant, the decrease of waist circumference between T0 and T1 of 8 mm was comparable between our diabetic and non-diabetic patients and also negligible, in contrast to the 31 mm decrease in waist circumference found for combined aerobic and resistance training [8]. In accordance to previous studies [17], the increase in VO_2_ peak in our study was not accompanied by a decrease in BMI.

### MACE

Similar to our study, DM was associated with increased risk for cardiovascular mortality but not for total mortality [60]. In contrast, total and cardiovascular mortality was found to be higher at 1 year after CR in another study [18]. Increased all-cause mortality and fatal and non-fatal cardiovascular disease in patients with uncontrolled DM has also been found in CAD patients of the EUROASPIRE IV study [61]. The pathophysiological link between DM and increased cardiovascular mortality have been suggested to be hyperglycaemia, as it exerts a direct effect on endothelial function and on the induction and progression of atherosclerosis, hyperinsulinaemia, insulin resistance, dyslipidaemia, inflammation, reactive oxygen species, endothelial dysfunction, hypercoagulability, and vascular calcification [62]. These may lead to heart failure, ACS or stroke [63]. Reductions in all-cause mortality by CR has been demonstrated also in patients with DM [64]. However, according to the LOOK AHEAD lifestyle intervention study in patients with type 2 DM, MACE has been found to be reduced in patients who lost at least 10% of their body weight and was independent of changes in exercise capacity, highlighting the importance of weight loss in these patients [65].

### Limitations

The biggest limitation of the present study is the lack of a control group not undergoing CR, which precludes any conclusions about the benefit of CR itself on outcomes. Glycemic control could only be estimated from HbA1c measurements, which may not well reflect changes over CR for those centres with short CR duration. However, also when the mixed model for HbA1c was adjusted for time after index event, results were comparable with only insulin therapy and greater BMI being associated with higher HbA1c. Last but not least, patients with type I diabetes were underrepresented in our sample.

## Conclusions

While immediate improvements in VO_2_ peak after CR in elderly patients with and without DM were similar, 12-month maintenance of this improvement was inferior in patients with DM, possibly related to disease progression. Glycemic control was less favourable in diabetic patients needing insulin in the short and long-term. Since glycemic control was only improved by weight loss but not by increase in exercise capacity, this highlights the importance of weight loss of obese DM patients during CR [13, 14, 16, 27].

## Supplementary information

**Additional file 1: Table S1**: Estimates and standard error of mixed models for peak VO_2_ [ml/kg/min] with patients as random intercept and time (end of CR and 1-year follow-up), age, sex, BMI, comorbidities and cardiovascular risk factors as fixed effects (Model 1). Diabetes mellitus and time interaction was also entered as fixed effect. Model 2 also included days after index event, resulting in different estimates for time points.

## Data Availability

The datasets generated and analysed during the current study are not publicly available due to restricting patient privacy regulations by the different countries but are available from the corresponding author on reasonable request.

## Abbreviations

ACS: Acute coronary syndrome
BMI: Body mass index
BP: Blood pressure
CABG: Coronary artery bypass grafting
CAD: Coronary artery disease
CR: Cardiac rehabilitation
DM: Diabetes mellitus
HbA1c: Glycated haemoglobin
HDL-C: High-density lipoprotein cholesterol
LDL-C: Low-density lipoprotein cholesterol
MACE: Major adverse cardiac event
MET: Metabolic equivalent of task
PCI: Percutaneous intervention
T2DM: Type 2 diabetes mellitus
VE/VCO_2_: Ventilatory efficiency (ventilation/rate of carbon dioxide production)
VO_2_: Rate of oxygen consumption
