# Supplementary material for: Clinical outcomes after cardiac rehabilitation in elderly patients with and without diabetes mellitus: The EU-CaRE multicenter cohort study
Source: Cardiovasc Diabetol. 2020 Mar 19;19:37. doi: 10.1186/s12933-020-01013-8 (PMC7081600; doi:10.1186/s12933-020-01013-8)
Supplement: Supplementary file 1 — Additional file 1: Table S1: Estimates and standard error of mixed models for peak VO2 [ml/kg/min] with patients as random intercept and time (end of CR and 1-year follow-up), age, sex, BMI, comorbidities and cardiovascular risk factors as fixed effects (Model 1). Diabetes mellitus and time interaction was also entered as fixed effect. Model 2 also included days after index event, resulting in different estimates for time points. [file 12933_2020_1013_MOESM1_ESM.docx]

**Table S1**: Estimates and standard error of mixed models for peak VO2 [ml/kg/min] with patients as random intercept and time (end of CR and 1-year follow-up), age, sex, bmi, comorbidities and cardiovascular risk factors as fixed effects (Model 1). Diabetes mellitus and time interaction was also entered as fixed effect. Model 2 also included days after index event, resulting in different estimates for time points.

|  | Model 1 |  |  | Model 2 |  |  |
| --- | --- | --- | --- | --- | --- | --- |
| Parameter | Estimate | SE | p-value | Estimate | SE | p-value |
| Intercept | 14.69 | 0.30 | 0.0000 | 15.63 | 0.33 | 0.0000 |
| End of CR | 2.01 | 0.09 | 0.0000 | 1.51 | 0.12 | 0.0000 |
| 1-year follow-up | 2.72 | 0.10 | 0.0000 | 0.01 | 0.41 | 0.9711 |
| Age [y] (centred) | -0.22 | 0.02 | 0.0000 | -0.22 | 0.02 | 0.0000 |
| BMI [kg/m^2^] (centred) | -0.29 | 0.02 | 0.0000 | -0.30 | 0.02 | 0.0000 |
| Male sex | 3.69 | 0.24 | 0.0000 | 3.71 | 0.24 | 0.0000 |
| Diabetes mellitus (DM) | -1.46 | 0.25 | 0.0000 | -1.44 | 0.25 | 0.0000 |
| Previous ACS | -1.00 | 0.26 | 0.0002 | -1.04 | 0.26 | 0.0001 |
| Nephropathy | -1.95 | 0.38 | 0.0000 | -2.01 | 0.37 | 0.0000 |
| PAD | -1.83 | 0.38 | 0.0000 | -1.79 | 0.38 | 0.0000 |
| Hypertension | -1.05 | 0.22 | 0.0000 | -1.03 | 0.22 | 0.0000 |
| Hypercholesterinaemia | 0.90 | 0.22 | 0.0000 | 0.87 | 0.22 | 0.0001 |
| *Index intervention (reference=PCI)* |  |  |  |  |  |  |
| Coronary artery bypass grafting | -1.65 | 0.23 | 0.0000 | -1.55 | 0.22 | 0.0000 |
| Surgical valve replacement | -2.22 | 0.38 | 0.0000 | -2.05 | 0.37 | 0.0000 |
| Percutaneous valve replacement | -0.29 | 0.71 | 0.6795 | -0.26 | 0.70 | 0.7154 |
| Stable angina | -0.35 | 0.44 | 0.4282 | -0.38 | 0.44 | 0.3834 |
|  |  |  |  |  |  |  |
| DM * end of CR interaction | -0.19 | 0.18 | 0.3128 | -0.20 | 0.18 | 0.2793 |
| DM * 1-year follow-up interaction | -0.60 | 0.19 | 0.0019 | -0.60 | 0.19 | 0.0019 |
| Days since index event (centred) |  |  |  | 0.007 | 0.001 | 0.0000 |
